# DPDDI: a deep predictor for drug-drug interactions

**DOI:** 10.1186/s12859-020-03724-x

**Published:** 2020-09-24

**Authors:** Yue-Hua Feng, Shao-Wu Zhang, Jian-Yu Shi

**Affiliations:** 1 Key Laboratory of Information Fusion Technology of Ministry of Education, School of Automation, Northwestern Polytechnical University, Xi’an, 710072 China; 2 School of Life Sciences, Northwestern Polytechnical University, Xi’an, 710072 China

**Keywords:** Drug-drug interaction, DDI prediction, Graph convolution network (GCN), Feature extraction, Deep neural network

## Abstract

**Background:**

The treatment of complex diseases by taking multiple drugs becomes increasingly popular. However, drug-drug interactions (DDIs) may give rise to the risk of unanticipated adverse effects and even unknown toxicity. DDI detection in the wet lab is expensive and time-consuming. Thus, it is highly desired to develop the computational methods for predicting DDIs. Generally, most of the existing computational methods predict DDIs by extracting the chemical and biological features of drugs from diverse drug-related properties, however some drug properties are costly to obtain and not available in many cases.

**Results:**

In this work, we presented a novel method (namely DPDDI) to predict DDIs by extracting the network structure features of drugs from DDI network with graph convolution network (GCN), and the deep neural network (DNN) model as a predictor. GCN learns the low-dimensional feature representations of drugs by capturing the topological relationship of drugs in DDI network. DNN predictor concatenates the latent feature vectors of any two drugs as the feature vector of the corresponding drug pairs to train a DNN for predicting the potential drug-drug interactions. Experiment results show that, the newly proposed DPDDI method outperforms four other state-of-the-art methods; the GCN-derived latent features include more DDI information than other features derived from chemical, biological or anatomical properties of drugs; and the concatenation feature aggregation operator is better than two other feature aggregation operators (i.e., inner product and summation). The results in case studies confirm that DPDDI achieves reasonable performance in predicting new DDIs.

**Conclusion:**

We proposed an effective and robust method DPDDI to predict the potential DDIs by utilizing the DDI network information without considering the drug properties (i.e., drug chemical and biological properties). The method should also be useful in other DDI-related scenarios, such as the detection of unexpected side effects, and the guidance of drug combination.

## Background

By taking advantage of the synergistic effects caused by drug-drug interactions (DDIs), the combinational treatment of multiple drugs for complex diseases are popular nowadays [1]. However, unexpected DDI can also trigger side effects, adverse reactions, and even serious toxicity, leading patients in danger [2]. As there exists increasing needs of multi-drug treatments, the identification of DDIs is more and more urgent. Nevertheless, it is expensive and time-consuming to detect DDIs among a large scale of drug pairs both in vitro and in vivo. To assist the screening of DDIs, computational approaches have been developed to deduce candidate drug-drug interactions.

Existing computational methods can be roughly classified into two categories: text mining-based and machine learning-based methods. The text mining-based methods discover and collect annotated DDIs from scientific literatures, electronic medical records [3, 4], insurance claim databases and the FDA Adverse Event Reporting System [5]. They are quite useful in building DDI-related databases. However, those methods cannot detect unannotated DDIs, and cannot give alerts to potential DDIs before a combinational treatment is made [2]. In contrast, machine learning-based methods provide a promising way to identify unannotated potential drug-drug interactions for downstream experimental validations.

Usually, machine learning-based methods consist of the feature extractor and the supervised predictor. The feature extractor represents drugs in a form of feature vector according to drug properties, such as chemical structure [2, 6–14], targets [2, 8–11], Anatomical Therapeutic Chemical classification (ATC) codes [8–10, 12], side effects [8, 9, 11, 13, 14], medication and/or clinical observations [11].

The supervised predictor is usually implemented by classification algorithms, such as KNN [12], SVM [12], logistic regression [2, 8, 10], decision tree [10], naïve Bayes [10]), and network propagation methods, such as reasoning over drug-drug network structure [6–8], label propagation [13], random walk [11, 15], probabilistic soft logic [9, 10]) or matrix factorization [14]. Usually, the predictor first trains a model with both feature vectors/similarity matrices and annotated DDI labels, then deduces potential DDIs with the well-trained model. Most methods utilize a single predictor [2, 5–8, 13–16], while some of them integrate multiple predictors [10, 12].

In general, the performance of existing approaches heavily relies on the quality of handcrafted features derived from the drug properties. However, some drug properties may not always be available. One common solution is to remove the drugs that lack a certain drug property, which results in small-scale pruned datasets and thus is not pragmatic and suitable in the real scenario [17]. In addition, some handcrafted drug features may not be precise enough to represent or characterize the property of drugs, which may jeopardize the construction of a robust and accurate model for link prediction.

As one of the most popular graph embedding methods, Graph Convolution Network (GCN) provides a promising way to predict DDIs when some properties of drugs are not available. Inspired by the traditional convolutional neural networks (CNNs) operating on regular Euclidean data like images (2D grid) and text (1D sequence) [18], GCN formulates convolution on an irregular graph in non-Euclidean domains, then aggregates information about each node’s neighborhood to distill the network into dense vector embedding without requiring manual feature engineering [19]. The dense vector embedding, also called low-dimensional representations, are learned to preserve the structural relationships between nodes (e.g., drugs) of the network, and thus can be used as features in building machine learning models for various downstream tasks, such as link prediction [17]. Recently, the GCN has been applied to the field of drug development and discovery [20], such as molecular activity prediction [21], drug side effect prediction [22], drug target interactions prediction [23].

In this work, we introduced a deep predictor of drug-drug interactions (namely DPDDI), which uses a graph convolution network (GCN) to learn the low-dimensional feature representation of each drug in the DDI networks, and adopts the deep neural network (DNN) to train models. GCN characterizes drugs in a graph embedding space for capturing the topological relationship to their neighborhood drugs. Experiment results demonstrate that our DPDDI outperforms other existing state-of-art methods in DDI prediction.

## Results

In this section, we first introduce how to set the parameters of DPDDI predictor, then compare the performance of DPDDI with four other state-of-the-art methods in 5-fold cross-validation (5CV) test. We also compare the results of three feature aggregation operators, discussing the effect of sampling rate of negative samples and the robustness of DPDDI on different scale dataset. In the end, we show the effectiveness of DPDDI through a case study.

In statistical prediction, the jackknife test and *q*-fold cross-validation (CV) test are often used to examine the effectiveness of a predictor [24]. Of the two test methods, the jackknife test is deemed the least arbitrary that can always yield a unique result [25]. However, for large scale database, the jackknife test is quite time consuming. To reduce the computational time and evaluate performance of a predictor, in this study, we adopted the 5-fold cross-validation (5CV) test as done by most investigators [26–29]. For 5CV test, the samples in the DDI dataset are randomly partitioned into 5 subsets with approximately equal size. One of the 5 subsets is singled out in turn as testing set; 90 and 10% of the other 4 subsets are used as the training samples (forming training set) and validation samples (forming validation set), respectively. The predictor is constructed on the training set and its parameters are tuned by using the validation set. This process is repeated for 5 iterations, each time setting aside a different test subset. To avoid the bias aroused from random data split, we implement 10 independent runs of 5CV, and use the average of the results to assess the performance of our DPDDI predictor.

### Parameter setting

We performed a grid search of the parameters by seeking both the minimum value of the loss function and the best accuracy with the training dataset. Both the GCN-based feature extractor and the DNN-based predictor need to tune the learning rate, epochs, batch size, dropout rate, as well as neuro numbers (dimensions) in hidden layers.

Specifically, with the full batch size, the GCN-based feature extractor tuned the learning rate (L-rate) from the list of {0.1, 0.01, 0.001, 0.005, 0.0001}, the Epochs from {200, 500, 800, 1000, 1200, 1400, 1600}, the Dropout from {0.01, 0.001, 0.0001}, and the hidden layer dimensions (H-dim) from {[800,512], [800,256], [800,128], [512,256], [512,128], [512,64], [256,64], [128,32]}. The DNN-based predictor tuned the learning rate (L-rate) from {0.1, 0.05, 0.01, 0.005}, the Epochs from {20, 40, 60, 80,100,140,160}, the batch size (B-size) from {10, 20, 40, 50, 60, 80}, the Dropout from {0.01, 0.001, 0.0001} and the hidden layer dimensions (H-dim) from {[128, 32], [128, 64], [64, 32], [128,64,32], [128, 32, 16], [64, 32, 16], [128, 64, 32, 16], [64, 32, 16, 4]}. The parameters led optimal prediction are shown in Table 1.

**Table 1:** The optimal parameters of DPDDI

Parameters	L-rate	Epochs	Dropout	B-size	I-dim^a^	H-dim	O-dim^b^
Feature extractor	0.001	1400	0.0001	Full-batch	1562	[512,128]	128
Predictor	0.01	140	0.001	50	256	[128,64,32]	2

^a^I-dim denotes the neuro numbers in input layer; ^b^O-dim denotes the neuro numbers in output layer

### Comparison with other state-of-the-art methods

We compared our DPDDI with four other state-of-the-art methods, including two Vilar’s methods (named as Vilar 1 and Vilar 2, respectively) [6, 7], label propagation-based method (named as LP) [13] and Zhang’s method (named as CE) [11] in 5-CV test. Vilar et al [6] integrates a Tanimoto similarity matrix of molecular structures with known DDI matrix by a linear matrix transformation to identify potential DDIs. Vilar et al [7] uses the drug interaction profile fingerprints (IPFs) to measure similarity for predicting DDIs. Label propagation method [13] applies label propagation to assign labels from known DDIs to previously unlabeled nodes by computing drug similarity-derived weights of edges on the DDI network. Zhang et al [11] collects a variety of drug-related data (e.g., known drug-drug interactions, drug substructures, proteins, pathways, indications, and side effects, etc.) to build many base classifiers, then performed the prediction with an ensemble (CE) classifier model.

To ensure a fair comparison, the DB2 dataset from [11] is adopted. In the DB2 dataset, all unlabeled drug pairs are considered as the negative samples. The comparison results in 5CV test are shown in Table 2, from which we can see that DPDDI achieves the best results, outperforming the other four state-of-the-art methods across all the metrics. Specifically, DPDDI achieves the improvements of 0.2 ~ 24.9%, 6.6 ~ 64.5%, 2.2 ~ 31.5%, 2.5 ~ 50.1%, 0.6 ~ 22.1%, 8.9 ~ 50.6% against other three methods of Vilar 1, Vilar 2 and LP in terms of AUC, AUPR, Recall, Precision, Accuracy, and F1-score, respectively. Although the AUC and ACC of DPDDI are slightly lower than that of Zhang’s method [11], the AUPR and *F*_1_ of DPDDI are higher. AUPR is often believed to be a more significant quality measure than AUC, as it punishes much more the existence of false positive drug-drug interactions. *F*_1_ represents the harmonic mean of precision and recall, which focus on the proportion of correctly predicted drug-drug interaction pairs. ACC focuses not only on the proportion of correctly predicted drug-drug interaction pairs, but also on the proportion of correctly predicted drug-drug non-interaction pairs. For the prediction of drug-drug interaction, *F*_1_ should be more effective measure than ACC.

**Table 2:** Performance comparison on DB2 dataset in 5CV test

Method	AUC	AUPR	Recall	Precision	ACC	*F*_1_
Vilar 1 [6]	0.707	0.262	0.495	0.253	0.719	0.334
Vilar 2 [7]	0.826	0.533	0.569	0.515	0.862	0.540
LP [13]	0.851	0.799	0.685	0.729	0.809	0.706
CE^a^ [11]	**0.957**	0.807	0.785	0.670	0.955	0.723
DPDDI	0.956	**0.907**	**0.810**	**0.754**	**0.940**	**0.840**

^a^The results are taken from Table 5 in Ref. [11]

In addition, Zhang et al [11] used 9 drug-related data sources, while our DPDDI just use the known drug-drug interaction data. If we integrate more drug-related data sources (e.g., drug substructure, drug target, drug enzyme, drug transporter, drug pathway, drug indication, drug side effect and drug off side effect used in [11]) to construct the dug-drug similarity network, using DPDDI framework to predict DDIs, DPDDI should be able to achieve better performance.

### Comparison of different feature aggregate operators

After obtaining the latent feature vectors of single drugs in the embedding space by GCN, we adopt three feature operators (i.e., inner product, summation and concatenation) to aggregate the feature vectors of two drugs into one feature vector for representing the drug-drug pairs. Then these aggregation feature vectors are fed into the DNN model to evaluate their effects to our DPDDI on DB1 dataset in 5CV test. As shown in Table 3, the concatenate operator achieves the best results and is thus selected in our DPDDI model to aggregate the feature vectors of drugs.

**Table 3:** Results of three feature aggregation operators on DB1 dataset

Operators	AUC	AUPR	Recall	Precision	ACC	*F*_1_
Inner product	0.938	0.810	0.709	0.761	0.927	0.734
Summation	0.970	0.898	0.774	0.854	0.949	0.812
Concatenation	**0.983**	**0.925**	**0.844**	**0.836**	**0.955**	**0.840**

### Comparison of the network structure features, chemical features and biological features of drugs

In order to evaluate the effectiveness of the network structure (NS) features, we also considered the chemical and biological features derived from three heterogeneous sources, such as chemical structure (CS), drug-binding proteins (DBP), and Anatomical Therapeutic Chemical Classification labels (ATC). Chemical structures of the drugs are characterized by 881-dimensional PubChem fingerprints. The DBP features of drugs are represented by 1121-dimensional binary vectors in which each bit indicates the binding occurrence of a specific DBP across the drugs. The 118-dimensional ATC features of drugs are converted from the 7-bit ATC code via a one-hot coding. These features (i.e., network structure features, chemical structure features, DBP features and ATC features of drugs) are respectively concatenated to feed the DNN models for predicting DDIs, and the results of these features with DNN on DB1 dataset in 5CV test are shown in Table 4, from which we can see that the network structure features generate the best performance.

**Table 4:** Comparing different types of features on DB1 data in 5CV test

Feature	AUC	AUPR	Recall	Precision	ACC	*F*_1_
CS	0.904	0.635	0.668	0.554	0.876	0.605
DBP	0.874	0.616	0.602	0.584	0.882	0.593
ATC	0.901	0.656	0.659	0.576	0.882	0.615
NS	**0.983**	**0.925**	**0.844**	**0.836**	**0.955**	**0.840**

### Influence of dataset scale size

In order to verify the robustness of our DPDDI approach, we use three datasets (i.e., DB1, DB2 and DB3) with different sizes to assess the performance of DPDDI in 5CV test. DB1 dataset contains 1562 drugs and 180,576 annotated drug-drug interactions. DB2 contains 548 drugs and 48,584 annotated drug-drug interactions. DB3 dataset contains 1934 drugs and 230,887 annotated drug-drug interactions. As shown in Table 5, although the dataset size has some effect on the performance of DPDDI (i.e., higher performance is achieved on dataset of a larger size), our DPDDI obtain reasonable prediction results on small dataset as well. These results show that our DPDDI approach is relatively robust with respect to the size of datasets for predicting DDI.

**Table 5:** Results of DPDDI on datasets of different size in 5CV test

Dataset	Type	Sparsity	AUC	AUPR	Recall	Precision	ACC	*F*_1_
DB2	Small	32.4%	0.956	0.907	0.810	0.754	0.940	0.840
DB1	Medium	14.8%	0.983	0.925	0.844	0.836	0.955	0.840
DB3	Large	12.4%	0.981	0.932	0.835	0.876	0.960	0.855

We also investigate the effects of negative sample size on DPDDI by sampling different unlabeled drug pairs to generate the negative sample sets, which are combined with the known DDI pairs (i.e., positive sample set) to form the DDI training, validation and testing datasets.

**Fig. 1:**
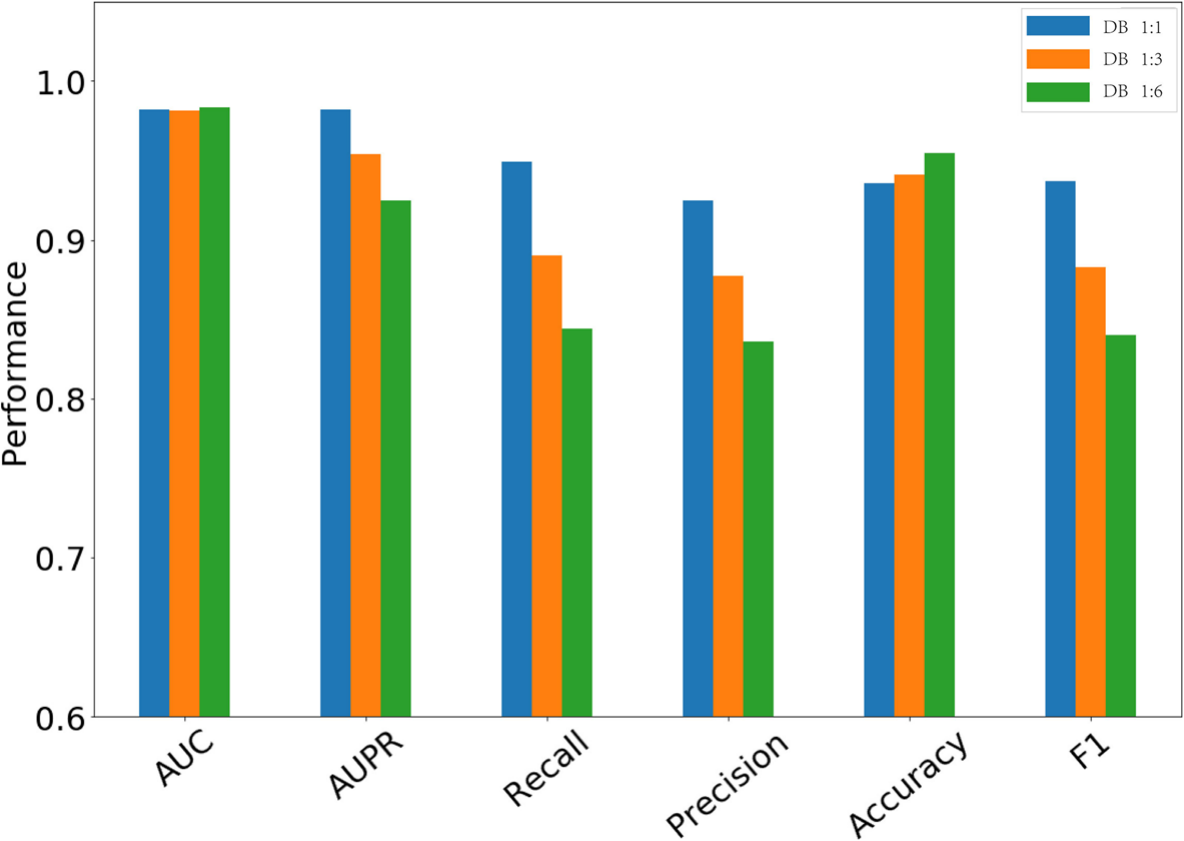
Impact of sample balancing

From DB1 dataset, we randomly selected different number of unlabeled drug pairs and combine them with the known DDI pairs to construct the datasets of DB1:1, DB1:3 and DB1:6, in which the ratio of positive samples (i.e., known DDI pairs) and negative samples (i.e., unlabeled drug pairs) are kept 1:1, 1:3 and 1:6, respectively. Figure 1 shows the results of DPDDI on DB1:1, DB1:3 and DB1:6 datasets in 5CV test. We can see that DPDDI achieves the highest values in terms of AUC, AUPR, Precision, Recall, Accuracy and *F*_1_ on DB1:1 dataset, indicating that the imbalance between positive and negative samples does have impacts on the performance of DPDDI.

### Case studies

In this section, we investigate the performance of DPDDI in predicting the unobserved DDIs. DB1 contains 180,576 annotated drug-drug interaction pairs among 1562 drugs, and 1,038,565 unlabeled drug pairs which may contain unobserved DDIs. By training DPDDI with DDI network from DB1 dataset, the possible interactions among drugs are inferred. Higher scores of unobserved drug pairs indicate that there are higher probabilities to interact between these drugs. Table 6 shows the top 20 predicted drug-drug interactions of DPDDI, which are not available in DB1 dataset. By searching for the evidence of these newly predicted DDIs on DrugBank (version 5.0) database and Drug Interaction Checker website (Drugs.com), we find that a significant fraction of newly predicted DDIs (13 out of 20) is confirmed. For instance, the description of the interaction between drug “Doxycycline” and drug “Bleomycin” is “Doxycycline may decrease the excretion rate of Bleomycin which could result in a higher serum level”. The case studies demonstrate that our DPDDI can effectively detect the potential drug-drug interactions. Maybe other 7 newly predicted DDIs our of 20 are confirmed by later experiments.

**Table 6:** Top 20 predicted DDIs by DPDDI

Number	Drug 1	Drug 2	Validation source	Description
1	Doxycycline	Bleomycin	DrugBank	Doxycycline may decrease the excretion rate of Bleomycin which could result in a higher serum level.
2	Doxycycline	Rifapentine	N/A	N/A
3	Doxycycline	Fusidic acid	N/A	N/A
4	Pramipexole	Paroxetine	DrugBank	Paroxetine may increase the sedative activities of Pramipexole.
5	Luliconazole	Doxycycline	N/A	N/A
6	Netupitant	Doxycycline	DrugBank	The metabolism of Netupitant can be decreased when combined with Doxycycline.
7	Tenoxicam	Minocycline	N/A	N/A
8	Etoperidone	Tenoxicam	DrugBank	Tenoxicam may decrease the excretion rate of Etoperidone which could result in a higher serum level.
9	Pramipexole	Minocycline	N/A	N/A
10	Ropinirole	Pramipexole	DrugBank	Ropinirole may increase the sedative activities of Pramipexole.
11	Minocycline	Ropinirole	DrugBank	Minocycline may increase the central nervous system depressant (CNS depressant) activities of Ropinirole.
12	Bleomycin	Doxycycline	DrugBank	Doxycycline may decrease the excretion rate of Bleomycin which could result in a higher serum level.
13	Pramipexole	Metyrosine	drugs.com	Using metyroSINE together with pramipexole may increase side effects such as dizziness, drowsiness, confusion, and difficulty concentrating.
14	Osimertinib	Doxycycline	DrugBank	The metabolism of Osimertinib can be decreased when combined with Doxycycline.
15	Dronabinol	Pramipexole	DrugBank	Dronabinol may increase the sedative activities of Pramipexole.
16	Rufinamide	Tenoxicam	N/A	N/A
17	Phenobarbital	Pramipexole	drugs.com	Using PHENobarbital together with pramipexole may increase side effects such as dizziness, drowsiness, confusion, and difficulty concentrating.
18	Bleomycin	Mitotane	N/A	N/A
19	Fosaprepitant	Doxycycline	DrugBank	The metabolism of Fosaprepitant can be decreased when combined with Doxycycline.
20	Duloxetine	Rufinamide	drugs.com	Using DULoxetine together with rufinamide may increase side effects such as dizziness, drowsiness, confusion, and difficulty concentrating.

In addition, among the top 20 predicted DDIs of DPDDI, we find that the drug of “doxycycline” interacts with other 8 drugs, and 5 out of 8 DDI pairs have been confirmed by current experimental evidences. These results indicate that “doxycycline” drug may have higher activity and is easy to interact with other drugs for implementing the drug efficacy.

## Discussions

One the key factor in DDI prediction is the features considered. We compared the GCN-derived DDI network structure feature with the other three chemical structure and biological features. The results in Table 4 show the superiority of our GCN-derived DDI network structure feature across all the performance metrics. Especially, our GCN-derived DDI network structure feature achieves > 20% improvement in terms of AUPR, Recall, Precision, and *F*_1_-score. These results demonstrate that DDI network structure features-based GCN contains more DDI discriminant information, and can effectively learn a low-dimensional feature representation for each drug in the DDI network, i.e., the low-dimensional representation preserve the ample structural information of DDI network.

In DDI prediction, how to best aggregate the feature vectors of two drugs into one vector for presenting one drug pair is another key factor. We adopt three feature operators of inner product, summation and concatenation to aggregate the feature vectors of two drugs. Results in Table 3 show that the concatenate operator achieves the best performance whereas the inner product operator gets the worst performance. Therefore, concatenation operator was adopted in our DPDDI.

In addition, we paid particular attention to how to balance samples in the training phase. Many former works in similar areas [22, 30, 31] adopted the same number of negative samples and positive samples to avoid the computational challenge caused by the sample imbalance. Consistently, our results in Fig. 1 show that the balanced sample scheme achieves the best performance in terms of AUPR, Recall, Precision and F1 score. These results indicate that the imbalance between positive and negative samples does have influence on DPDDI. For fairly comparing with other state-of-the-art methods, the known drug-drug interaction pairs (positive samples) and all unlabeled drug-drug pairs (negative samples) are used to train the prediction model. Considering that more sever sample imbalance can result in the higher errors, we also introduce a weight *W*_*pos*_ in Eq.(2) for sample balancing.

The comparison experiments (in Tables 1 and 4) demonstrate the superior performance and robustness of DPDDI compared to four other state-of-the-art methods on three DDI datasets with different scale. Investigation on the top predicted DDIs confirm the competence of DPDDI for predicting the new DDIs.

The superior performance of DPDDI can be attributed to the following aspects: i) Designing a GCN model to learn the low-dimensional feature representations of drugs and capture the structure information of DDI network. ii) Constructing a DNN model as the predictor to distinguish whether interaction exists between two drugs. iii) DNN model can learn the non-linear relationship of drug pairs by mapping the drug pairs from a high-dimension space into a lower dimension space.

DPDDI is effective in predicting the potential interactions between two drugs existed in DDI network. If the DDI network does not contain the drugs, e.g., a newly invented drug without prior information, DPDDI will fail. In this condition, it is possible to construct the drug-drug similarity network by introducing the drug chemical or biological properties, and then implement our DPDDI framework to predict the novel DDIs.

## Conclusions

Aiming at the preliminary screening of DDIs, this work presents a novel prediction method (namely DPDDI) from a DDIs network. DPDDI consists of a feature extractor based on graph convolution network (GCN) and a predictor based on deep neural network (DNN). The former characterizes drugs in a graph embedding space, where each drug is represented as a low-dimensional latent feature vector for capturing the topological relationship to its neighborhood drugs. The latter concatenates latent feature vectors of any two drugs into one feature vector to represent the corresponding drug pairs for train a DNN for predicting potential interactions. Designated experiments for DPDDI bring several observations: i) the concatenation feature aggregation operator is better than two other feature aggregation operators, i.e., the inner product and the summation; ii) the GCN-derived latent features greatly outperform other features derived from chemical, biological or anatomical properties of drugs; iii) DPDDI is robust to the datasets with different scale in drug number, DDI number, and network sparsity; iv) the performance of DPDDI is significantly superior to four state-of-the-art methods; v) the finding of 13 verified DDIs out of top 20 unobserved candidates in case studies reveals the capability of DPDDI for predicting new DDIs. To summarize, the proposed DPDDI is an effective approach for predicting DDIs, and should be helpful in other DDI-related scenarios, such as the detection of unexpected side effects, and the guidance of drug combination.

## Methods

### Datasets

We extracted the approved small molecular drugs and their interaction relationships from DrugBank 4.0 [32] to build the DB1 dataset which contains 1562 drugs and 180,576 annotated drug-drug interactions. In order to compare with other state-of-the-art methods, a smaller dataset (named as DB2) built by Zhang et al. [11] was adopted to evaluate the performance of our DPDDI. DB2 contains 548 drugs and 48,584 annotated drug-drug interactions. Moreover, we also collected a new and larger dataset from DrugBank 5.0 [33] to build the DB3 dataset for assess the robustness of our DPDDI, including 1934 drugs and 230,887 annotated drug-drug interactions. In DB1, DB2 and DB3, the known drug-drug interaction pairs are used as the positive samples to build the positive set, and the other unlabeled drug-drug pairs are considered as the negative samples in which we utilize a random sampling strategy to build the negative set. From the perspective of interactions, these three datasets can be treated as DDI networks. The network characteristics are summarized in Table 7.

**Table 7:** Characteristics of DDI networks from DB1, DB2 and DB3

Dataset	#Drug	#Interaction	#No-link	Sparsity	Max degree	Min degree
DB1	1562	180,576	1,038,565	14.8%	903	1
DB2	548	48,584	101,294	32.4%	512	1
DB3	1934	230,887	1,637,357	12.4%	1049	1

# denotes the number of drugs, link drug-drug pairs and no-link drug-drug pairs

In order to compare our network-based features with other drug features derived from diverse drug properties, we also downloaded the drug chemical structures, Anatomical Therapeutic Chemical classification (ATC) codes and drug-binding proteins (DBPs) from DrugBank.

The chemical structure-based feature represents each drug by an 881-dimensional binary vector in which each bit represents the specific substructure according to Pubchem fingerprints. ATC codes are released by the World Health Organization [34], and they categorize drug substances at different levels according to organs they affect, application area, therapeutic properties, chemical and pharmacological properties. It is generally accepted that compounds with similar physicochemical properties exhibit similar biological activity. As 138 of 1562 drugs in DB1 have no ATC code, we adopted their predicted codes by SPACE [35], which deduce ATC codes from chemical structures. To feed the 7-bit ATC code into DNN, we convert them into a one-hot code with 118 bits.

We also used drug-binding protein (DBP) data collected by [16], including 899 drug targets and 222 non-target proteins. Similarly, each drug is represented as a binary DBP-based feature vector, of which each bit indicates whether the drug binds to a specific protein.

### Problem formulation

Our task is to deduce DDI candidates among those unannotated drug-drug pairs based on annotated DDIs in the form of a network. Technically, let *G*(*D*, *E*) be a DDI network, where *D* = {*d*_1_, *d*_2_, …, *d*_*m*_} is the set of *m* approved drugs and *E* denotes the interactions between them. This network can be usually represented by an *m* × *m* symmetric binary adjacency matrix *A*_*m* × *m*_ = {*a*_*ij*_}, where *a*_*ij*_ = 1 indicates an annotated interaction between drug *d*_*i*_ and drug *d*_*j*_, and otherwise no annotated interaction between them.

DDI prediction can be solved by a three-step approach. First, the function of *f*_1_(*A*) is to obtain the latent feature vector *Z*_*i*_ of each drug in *A*, where *Z*_*i*_ ∈ *R*^1 × *k*^(*k* ≪ *m*) . Next, the latent vectors (*Z*_*i*_ and *Z*_*j*_) of two drugs are aggregated into one feature vector to represent a drug pair. Last, the function of *f*_2_(*Z*_*i*_, *Z*_*j*_) ( *Z*_*i*_, *Z*_*j*_ ∈ *Z*) is used to reconstruct the network $\hat{A}$. The function of *f*_1_ is referred as the feature extractor, while the function of *f*_2_ is named as the predictor in our model.

**Fig. 2:**
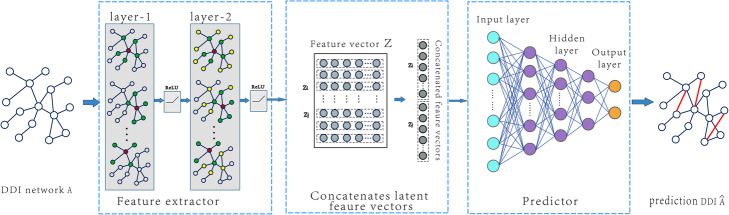
Overall framework of DPDDI. The main steps are as follows. First, the feature extractor of DPDDI constructs a two-layer graph convolutional network (GCN) to obtain drug latent features, which capture the complex relations between the drug nodes in the DDI network. Then, each pair of drugs is represented as a feature vector by concatenating the corresponding latent features of the drugs. Last, the feature vectors of representing the drug pairs are fed into a deep neural network to train the predictor to deduce potential DDIs

In this work, by implementing the solution based on deep learning, we provide a Deep Predictor for Drug-Drug Interactions (named as DPDDI). DPDDI mainly consists of the following three phases: i) Extract the low-dimensional embedding latent features of drugs from DDI network by building a GCN model; ii) Aggregate the latent feature vectors (i.e., *Z*_*i*_ and *Z*_*j*_) of drugs *d*_*i*_ and *d*_*j*_ to represent the drug pairs; iii) Feed the fused feature vectors into a DNN to predict DDIs. The overall framework of DPDDI is illustrated in Fig. 2.

The loss of DPDDI contains two parts as follows:

1 $$ Loss={L}_f\left(p,q\right)+{L}_p\left(p,q\right), $$

where *L*_*f*_ is the loss of its feature extractor, and *L*_*p*_ is the loss of its predictor. The first part adopts a binary weighted-cross-entropy as follows:

2 $$ {L}_f\left(p,q\right)=-{\sum}_{i,j}p\left({a}_{\mathrm{ij}}\right)\ \log \left(q\left({a}_{ij}\right)\right)\ast {W}_{pos}+\left(1-p\left({a}_{ij}\right)\right)\Big(1-\log \left(q\left({a}_{ij}\right)\right), $$

where *p*(*a*_*ij*_) is the true label of the training interaction *a*_*ij*_, $q\left({a}_{ij}\right)=\sigma \left({z}_i\bullet {z}_j^T\right)$ is the predicting probability computed by the inner product of latent vectors of two nodes generated by the GCN, and *W*_*pos*_ is the weight equal to the number of negative samples over the number of positive samples. The second part is defined by a binary cross-entropy as follows:

3 $$ {\mathrm{L}}_p\left(p,q\right)=-\sum \limits_{i,j}p\left({a}_{ij}\right)\ \log \left(s\left({a}_{ij}\right)\right), $$

where *s*(*a*_*ij*_) is the predicting probability generated by the DNN.

### Feature extractor

We employ a two-layer auto-encoder of graph convolutional network (GCN) [36, 37] to obtain embedding representations of drug nodes. Each drug is represented as a latent feature vector, which contains the high-dimensional information about its neighborhood in the DDI network without manual feature engineering. Such node embedding provides a promising way to represent the relationship between nodes in a complex network.

Technically, the GCN takes the adjacency matrix *A* as the input and outputs embedding vectors $\left\{{Z}_i\in {R}^{1\times {H}_p},i=1,2,\dots, m\right\}$ for every drug in the DDI network, where *H*_*p*_ is the dimension of the last hidden layer. Like [38] recommendation, our GCN adopts two layers as well. Suppose that *H*^(0)^ is the feature matrix in which each row denotes the input feature vector of each node in the network. In case of no input features, *H*^(0)^ is just an identity matrix. Then, the output *H*^(1)^ of the first hidden layer is defined as:

4 $$ {H}^{(1)}=f\left({H}^{(0)},A\right)=\mathrm{ReLU}\left(\hat{A}{H}^{(0)}{W}^{(0)}\right), $$

where $\hat{\mathrm{A}}={\overset{\sim }{\mathrm{D}}}^{-\frac{1}{2}}\overset{\sim }{\mathrm{A}}{\overset{\sim }{\mathrm{D}}}^{-\frac{1}{2}}$ is the symmetrically normalized adjacency matrix, ${\overset{\sim }{\mathrm{D}}}_{ii}={\sum}_j{\overset{\sim }{\mathrm{A}}}_{ij}$ and $\overset{\sim }{A}=A+{I}_N$, ${W}^{(0)}\in {R}^{m\times {H}_1}$ is the weight matrix to be learned, and ReLU is the activation function. Similarly, the output *H*^(2)^ of the second hidden layer is recursively defined as:

5 $$ {H}^{(2)}=f\left({H}^{(1)},A\right)=\mathrm{ReLU}\left(\hat{A}{H}^{(1)}{W}^{(1)}\right), $$

where ${W}^{(1)}\in {R}^{H_1\times {H}_2}$. Because our GCN contains only two layers, *H*^(2)^ is just the final embedding matrix *Z* $\in {R}^{m\times {H}_2}$.

### Feature aggregation for drug pairs

So far, the latent feature vector of single drug in the embedding space is obtained. The next task is to obtain feature vectors of drug pairs. Given two drugs *d*_*i*_ and *d*_*j*_, and their latent vectors ***Z***_*i*_ and ***Z***_*j*_ obtained by GCN, three feature operators, i.e., inner product, summation and concatenation, are considered to aggregate the latent feature vectors of two drugs into a single feature vector to represent the drug-drug pair. Specifically, we separately adopt the inner product $\boldsymbol{F}\left({d}_i,{d}_j\right)={\boldsymbol{Z}}_i\ {\boldsymbol{Z}}_{\boldsymbol{j}}^{\boldsymbol{T}}$, summation ***F***(*d*_*i*_, *d*_*j*_) = ***Z***_*i*_ + ***Z***_*j*_ and concatenation ***F***(*d*_*i*_, *d*_*j*_) = [***Z***_*i*_, ***Z***_*j*_] of two drug latent vectors *Z*_*i*_ and *Z*_*j*_ to represent the drug pair (*d*_*i*_, *d*_*j*_).

### Predictor

Given the feature vectors of drug-drug pairs, we construct a deep neural network (DNN) as the predictor in DPDDI for its the proven performance in classification. The predictor transforms DDI prediction into a binary classification, which is implemented by a five-layer DNN. The numbers of neurons in the layers of the DNN are 256, 128, 64, 32 and 2, respectively. ReLU is adopted as the activation function in the first four layers, while SoftMax is used as the activation function in the last layer, which outputs how likely drug pairs are potential DDIs.

There are two steps to train our DPDDI. The first step is to train a GCN for obtaining the low-dimensional embedding latent features of drugs. The parameters (i.e., learning rate, epochs, dropout, input-dim, hidden-dim, and output-dim) in GCN architecture are trained with the DDI network data. The second step is to learn the parameters (i.e., learning rate, dropout, epochs, batch-size, input-dim, hidden-dim, and output-dim) of the DNN for final DDI prediction and to fine turn all the parameters of DPDDI framework. To explain our DPDDI method in detail, the pseudo-code is shown in Table 8.

**Table 8:** The pseudo-code of DPDDI

**Input:** DDI network A	
The parameters: learning rate, epochs, dropout, batch-size, input-dim, hidden-dim, output-dim (both in Feature extractor and Predictor)	
**Output:** DDI network $\hat{\mathrm{A}}$ reconstructed by DPDDI	
1: Initialize parameter sets *W*^(0)^ and *W*^(1)^ in Feature extractor.	
2: Learn drug representations Z.	
3: **for** epoch **in** epochs (Feature extractor in Table 1.):	
4: Compute the loss function based on Eq. 2.	
5: Calculate gradient and adopt Adam optimizer to update *W*^(0)^ and *W*^(1)^.	
6: **end for**	
7: Obtain the representations Z of drugs according to Eq. 4 and Eq. 5.	
8: **for** each drug pair, do	
9: Feature aggregation by concatenating operation.	
10: **end for**	
11: Initialize parameter sets in Predictor based on DNN.	
12: Feed representation vector of each drug pair into Predictor.	
13: **for** epoch **in** epochs (Predictor in Table 1.):	
14: Compute the loss function based on Eq. 3.	
15: Calculate gradient and adopt Adam optimizer to update parameter sets .	
16: **end for**	
17: Obtain the DDI network $\hat{\mathrm{A}}$.	

### Evaluation metrics

The following metrics of accuracy (ACC), Recall, Precision and F_1_-score are used to measure the performance of DPDDI.

6 $$ \mathrm{Accuracy}=\frac{TP+ TN}{TP+ FP+ TN+ FN}, $$

7 $$ \mathrm{Precision}=\frac{TP}{TP+ FP}, $$

8 $$ \mathrm{Recall}=\frac{TP}{TP+ FN}, $$

9 $$ {F}_1=\frac{2\times Precision\times Recall}{Precision+ Recall}, $$

where *TP* and *TN* are the number of correctly predicted DDI pairs and unlabeled drug-drug pairs, respectively; *FP* and *FN* are the number of incorrectly predicted DDI pairs and unlabeled drug-drug pairs, respectively.

We also used the metrics of AUC and AUPR to measure the performance of our DPDDI. AUC is the area under the receiver operating characteristic (ROC) curve which illustrate the true-positive rate (i.e., *TP*/(*TP* + *FN*)) versus the false-positive rate (i.e., *FP*/(*FP* + *TN*)) at different cutoffs. AUPR is the area under the precision–recall curve which plots the ratio of true positives among all positive predictions for each given recall rate.

## Data Availability

The datasets generated and analyzed during the current study and the code of DPDDI are openly available at the website of https://github.com/NWPU-903PR/DPDDI.

## Abbreviations

DDIs: Drug-drug interactions
GCN: Graph convolution network
DNN: Deep neural network
ATC: Anatomical Therapeutic Chemical classification
DBP: Drug-binding protein
AUC: Area Under the receiver operating characteristic Curve
AUPR: Area Under the Precision-Recall curve
ACC: Accuracy
